# Structural and Photoelectric Properties of Epitaxially Grown Vanadium Dioxide Thin Films on c-Plane Sapphire and Titanium Dioxide

**DOI:** 10.1038/s41598-019-45806-8

**Published:** 2019-06-27

**Authors:** Jason A. Creeden, Scott E. Madaras, Douglas B. Beringer, Melissa R. Beebe, Irina Novikova, R. Ale Lukaszew

**Affiliations:** William & Mary Department of Physics, Williamsburg, Virginia 23187 USA

**Keywords:** Surfaces, interfaces and thin films, Sensors and biosensors, Electronic properties and materials

## Abstract

Vanadium dioxide (VO_2_) is one of the most extensively studied materials in the strongly correlated electron family capable of sustaining an insulator-to-metal transition. Here we present our studies of high-quality thin films of epitaxially grown VO_2_ on c-Al_2_O_3_(0001) and TiO_2_(001) via reactive DC pulsed magnetron sputtering. We present the structural transition probed via Reflection High Energy Electron Diffraction (RHEED) for the first time and we correlate the surface microstructure measurements with simulations before, during, and after the thermally induced transition. We also study the photoelectric conversion of VO_2_ on TiO_2_(001) and c-Al_2_O_3_(0001) under 405 nm light and demonstrate up to a 2000% increase in quantum efficiency as the power of the light is varied for VO_2_ on TiO_2_(001).

## Introduction

A Mott transition is a fundamental concept that governs the emergence of various electronic phases and physical properties in correlated electron materials, represented for example by the insulator-metal transition (IMT) in vanadium dioxide (VO_2_). Vanadium dioxide is one of the most extensively studied materials in the correlated electron family where it is known to undergo a characteristic reversible first order transition from insulator (T < T_c_) to metal (T > T_c_) where T_c_, ~68 °C in bulk, is the temperature at which the transition occurs^1,2^. This IMT is also accompanied by an associated structural transition from an insulating monoclinic phase to a metallic rutile phase^2^. Thus, it is subject of significant importance to understand the dynamics of electron and lattice systems across such transition in these highly correlated materials^3–5^. An interesting proposed aspect of VO_2_ in its monoclinic phase is its ability to undergo photoelectric conversion; where holes are formed in the valence band of the substrate and transported to the O_2p_ band of VO_2_ then electrons from the VO_2_ d_||_ band move to fill the holes in the lower O_2p_ band^6^. Therefore, a key approach to understand the occurrence of this photoelectric conversion is to investigate the correlations between surface microstructure and photoelectric conversion in thin films deposited on different substrates to explore the potential parameter space for possible applications.

Additionally, it has been argued that the binding between doubly occupied (doublon) and empty (holon) sites plays a key role in the Mott transition in strongly correlated Mott-Hubbard systems and could play a distinct role in photelectric conversion^7^. In this photo-electric conversion, the application of an electric field to the monoclinic VO_2_ phase drives carrier tunneling to create doublon-holon pairs via nonlinear excitation processes such as multiphoton absorption and quantum-tunneling^8,9^. This pair creation results in an instantaneous insulator to metal transition without direct interaction with the lattice^8,9^. Allowing for a purely electronic phase transition, however, the thin film surface microstructure and morphology will affect carrier scattering, and the dynamics will be reflected in the ultimate quantum-efficiency^9–11^.

## Results

### Crystal analysis and determination

**Figure 1:**
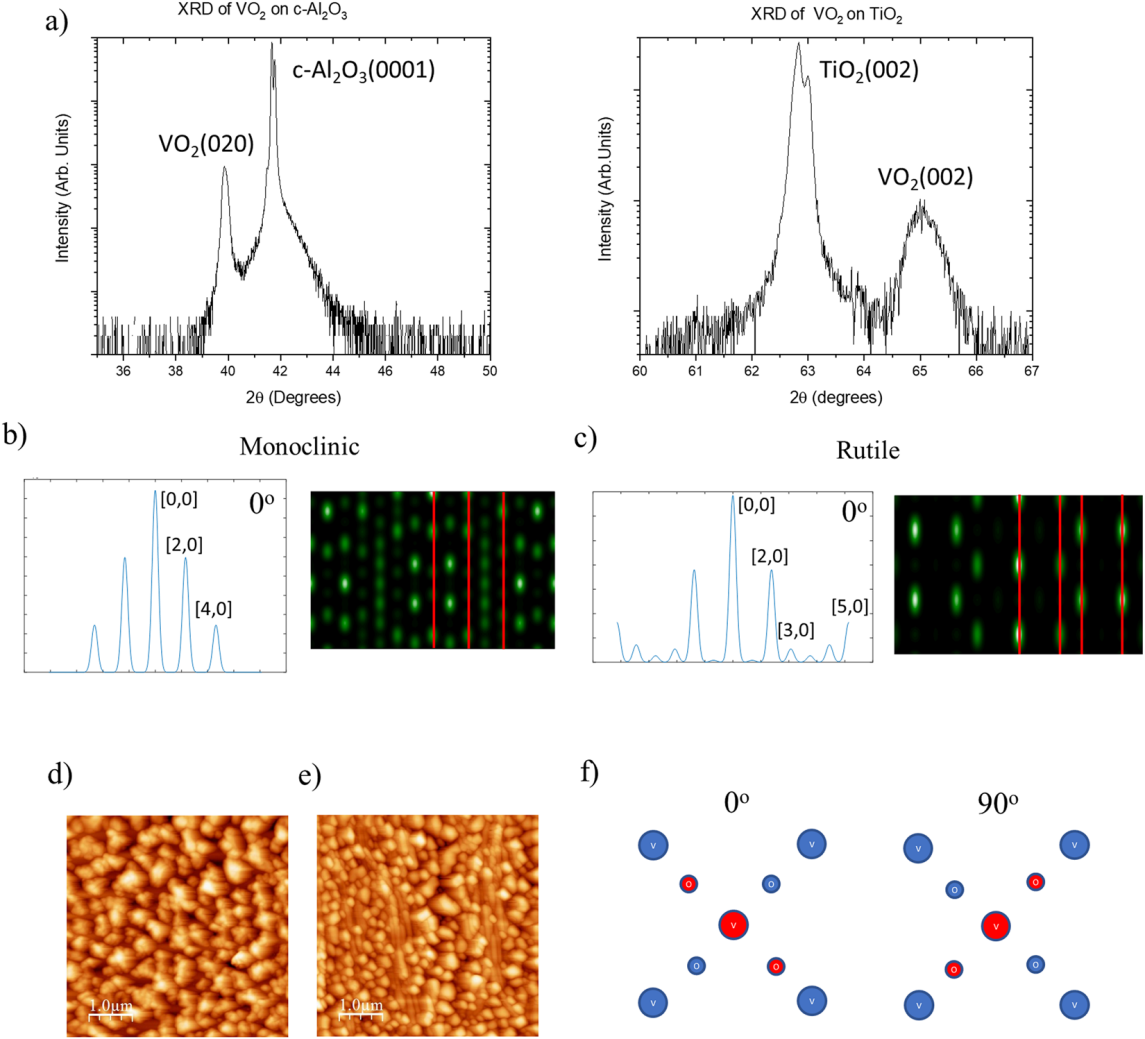
The experimental XRD, AFM, and simulated RHEED patterns of VO_2_ on c-Al_2_O_3_ and TiO_2_. (**a**) The XRD 2θ scan for VO_2_ on c-Al_2_O_3_(0001) and TiO_2_(001) where the intensity scale in arbitrary units is log scaled. (**b**) The simulated RHEED pattern of the 0° rotation for the monoclinic phase of VO_2_ where the streak intensity patterns are recorded on top and the simulated diagrams are reported on bottom with the in plane lattice planes are recorded for each. (**c**) The simulated RHEED patterns of the 0° rotation for the rutile phase of VO_2_ where the placement of the plots is the same as the previous monoclinic phase. (**d**) The AFM image of the surface microstructure of VO_2_ on c-Al_2_O_3_(0001) where the scale is 5.0 μm × 5.0 μm. (**e**) The AFM image of the of the surface microstructure of VO_2_ on TiO_2_(001) where the scale is 5.0 μm × 5.0 μm. (**f**) The orientations of the vanadium and oxygen atoms through the 0° and 90° rotations of one unit cell where the first two surface layers of atoms are shown in the c-direction where the red layer is displaced ~1.44 nm below the blue layer.

All films are 30 nm thick and were epitaxially grown via reactive DC magnetron sputtering on c-Al_2_O_3_ and TiO_2_ as discussed in the experimental section. The initial microstructure characterization of the films was carried out using X-Ray Diffraction (XRD) symmetric scans as shown in (Fig. 1a). Upon examination of the 2θ scans we determined that the peak location was consistent with the bulk location for the VO_2_ grown on c-Al_2_O_3_, with a slight degree of strain toward the substrate peak as expected. We note that the main peak location for the VO_2_ grown on TiO_2_ is consistent with previous reports for growth on this substrate^12^. For VO_2_ on TiO_2_(001), the VO_2_ peak location indicated that the film was also strained toward the substrate peak by ~0.5° in comparison to existing data, suggesting that in this case the film contains a more strained monoclinic phase toward the rutile phase than previously reported^12^.

We also determined the mosaicity (the degree of crystallite misorientation) for each sample and found a nominal degree of mosaicity ~0.08° for the sample grown on c-Al_2_O_3_ and ~0.047° for the sample grown on TiO_2_.

### RHEED structural analysis

Figure 1b,c shows the simulated Reflection High Energy Electron Diffraction (RHEED) patterns and streak diagrams expected for the bulk-like phases of VO_2_ through the IMT^13,14^. As evidenced by (Figs 1b,c,f and S1a–c), upon in-plane rotation of the sample with respect to the electron-beam, the diffraction pattern of the sample changes greatly due to the atomic positions of the vanadium and oxygen atoms on the lattice. The oxygen atoms play a distinct role in these diffraction patterns, as they appear to be the main contributors to streak intensity. Each in-plane rotation shows a large degree of variation in diffraction pattern, with a significant change expected for the monoclinic structure due to the canted angles exhibited by the oxygen atoms^15^.

The positions of the vanadium and oxygen atoms have been extensively studied both theoretically and experimentally for both phases across the IMT where space groups for the two phases of VO_2_ across the IMT are P4_2_/mmm(136) for rutile and P2_1_/c (14) for monoclinic and for our simulation atom positions were determined from these space groups as shown in Tables 1 and 2^15^. The rutile structure possesses greater in-plane symmetry than the monoclinic does. Thus, through each 90° in-plane azimuthal rotation a repetition of patterns will occur with a clear differentiation in diffraction patterns expected along the 45° rotation directions, and with differing patterns between 45° and 135°, depending upon whether the oxygen atoms are in-line with the main axis or rotated 90° off axis.

**Table 1:** Atomic Positions of VO_2_(R).

Atom	X(Å)	Y(Å)	Z(Å)
Vanadium	0	0	0
	2.275	2.275	1.44
Oxygen	1.38775	1.38775	0
	−1.38775	−1.38775	0
	0.88725	3.66275	1.44
	3.66275	0.88725	1.44
	1.38775	1.38775	2.88
	−1.38775	−1.38775	2.88

**Table 2:** Atomic Positions of VO_2_(M).

Atom	X(Å)	Y(Å)	Z(Å)
Vanadium	1.3915	5.2845	0.1345
	−1.3915	7.9945	2.5555
	−1.3915	−5.2845	−0.1345
	1.3915	−2.5745	2.8245
Oxygen 1	0.575	1.1382	1.076
	−0.575	3.8482	1.614
	−0.575	−1.1382	−1.076
	0.575	1.5718	3.766
Oxygen 2	2.2425	3.7398	1.5602
	−2.2425	6.4498	1.1298
	−2.2425	−3.7398	−1.5602
	2.2425	−1.0298	4.2502

This in-plane azimuthal dependency is illustrated in (Figs 1f and S2c) in which the first two layers of atoms account for the majority of the diffracted e^–^beam intensity in the patterns. For the present study, each of the chosen substrates ensures a good lattice match between the square lattice face of the substrates and the square face of the rutile structure for VO_2_ (a = 4.55 Å). In the case of c-Al_2_O_3_ the lattice mismatch is greater (a = 4.785 Å) than that of TiO_2_(001) (a = 4.584 Å) however both provide adequate lattice matching to facilitate epitaxial growth of VO_2_ as evidenced from the XRD in (Fig. 1a)^15–17^.

It is worth noting that the spotty nature of the simulated RHEED pattern (Figs 1b,c and S1a,b) is due to the fact that all surface atoms in the VO_2_ structure are not in the same plane, since all surface atoms in the same plane would lead to vertical streaks in the diffraction pattern. In the present case, this spotty pattern is compounded in the experimental RHEED data by the inherent surface roughness that is evidenced in the Atomic Force Microscopy (AFM) images of the actual samples (Fig. 1d,e). We also note that the surface of VO_2_ on c-Al_2_O_3_ has a larger degree of average roughness, ~30 nm, due to the larger spacing between terraces leading to a roughness on the order of the film thickness. In comparison, (Fig. 1e), corresponding to VO_2_ on TiO_2_(001), exhibits enhanced proximity between terraces, with each terrace sitting directly adjacent to its neighbor. This lack of separation between terraces accounts for a roughness ~13 nm thus yielding a smoother overall film in comparison to VO_2_ on c-Al_2_O_3_(0001). This larger degree of surface roughness in the VO_2_ on c-Al_2_O_3_(0001) may also be a contributor to the lesser photocurrent produced for the VO_2_ on c-Al_2_O_3_(0001) compared to the VO_2_ on TiO_2_(001) sample due to scattering effects discussed later.

**Figure 2:**
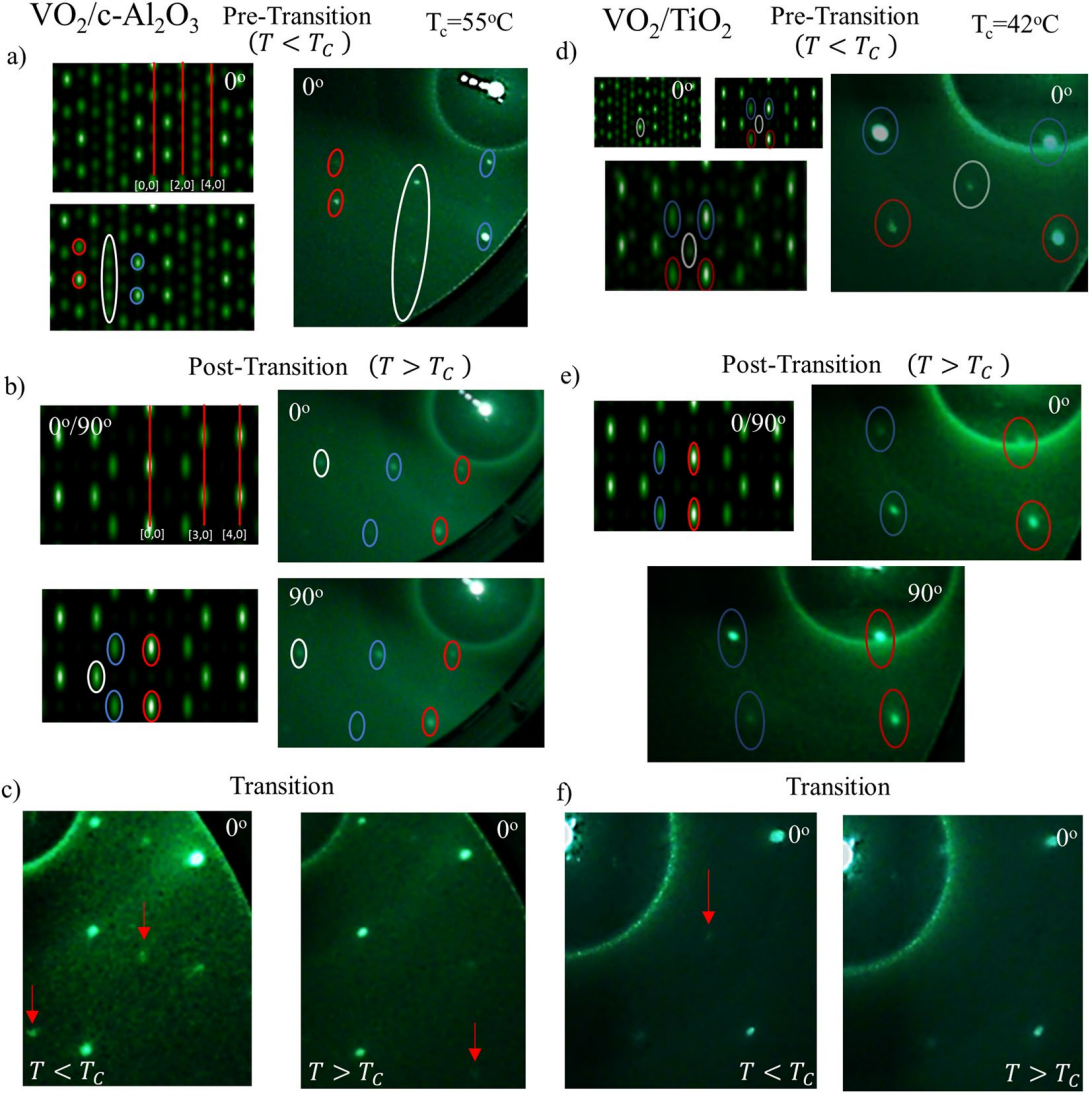
The simulated and experimentally determined RHEED patterns for VO_2_ on c-Al_2_O_3_(0001) and VO_2_ on TiO_2_(001). For VO_2_ on c-Al_2_O_3_(0001): Simulated and experimental RHEED patterns of VO_2_ azimuthal rotations and for the two phases. (**a**) The left two images are simulations of the 0° rotation for the monoclinic phase of VO_2;_ the top image denotes the 2-D lattice planes for the streak patterns while the bottom image has streaks highlighted. The right image is the experimentally determined RHEED pattern the highlighted streaks correspond to the simulated image. This plot orientation is maintained for (**b**) as well where the simulated images are shown at left and experimental images at right. (**b**) The 0°/90° rotation for the rutile phase of VO_2_. (**c**) The experimentally determined RHEED patterns for the 0° degree rotation through the thermal IMT where the image on the left is prior to transition and the image on the right is post transition. For VO_2_ on TiO_2_(001): (**f**) The top leftmost two images are simulations of the 0° rotation for the monoclinic phase (left) and rutile phase (right) of VO_2._ The bottom left image is a superimposed image of both simulated images. The right image is the experimentally determined RHEED pattern for VO_2_ on TiO_2_(001) where the highlighted streaks correspond to the simulated images. (**e**) The left top image is the simulation and right top and bottom images experimental patterns for VO_2_ grown on TiO_2_(001); the 0° rotation is right and 90° is bottom. (**f**) The experimentally determined RHEED patterns for the 0° degree rotation through the thermal IMT where the image on the left is prior to transition and the image on the right is post transition. (Note: All images have been rotated 45 degrees and the contrast has been increased for ease of streak identification).

We also present here a comparison study of bulk VO_2_ RHEED patterns predicted via simulation and the RHEED patterns recorded from VO_2_ grown on c-Al_2_O_3_. As shown in (Figs 2a and S2a) the experimentally determined RHEED patterns exhibit similar streak configurations and orientations as those determined in the simulated patterns. Figure S2a exhibits numerous cases of pattern matching as well as streak spacing suggesting that the simulations and the observed diffraction patterns for the monoclinic VO_2_ phase are in good agreement. We then proceeded to heat the samples up to 100 °C to allow for the VO_2_ to undergo the full IMT which for this sample occurred at ~55 °C. This shift in transition temperature from bulk is likely due to the thin film character of sample, where factors like lattice parameter mismatch between sample and substrate play a key role as previously reported for TiO_2_^12,18^. Once the sample fully transitioned, the RHEED patterns in (Figs 2b and S2b) (right) were compared to the simulations shown in (Figs 2b and S2b) (left). The streak patterns from the rutile phase VO_2_ exhibit the same configurations as those predicted in the simulation for (Figs 2b and S2b), i.e. experimentally determined patterns are similar to the simulated ones, in combination with the AFM images and the XRD data, further assert that the patterns observed in both the simulations and experiments, are quite close to bulk-like crystalline VO_2_ in both the monoclinic and rutile states, and with low mosaicity.

We also examined the RHEED pattern through the transition to investigate the sample evolution in real time. To do so, we heated the sample in 5 °C increments from 25 °C to 100 °C recording the diffraction pattern after each increment. Upon ramping the temperature, we found a marked change in the RHEED pattern at the critical temperature. We observed the disappearance of several peripheral streaks exhibited in the monoclinic phase as well as a strengthening of the intensity of the main streaks that create a repeating “rectangular” pattern as designated by (Fig. 2c and Supplementary Video S1). We propose that this change in diffraction pattern is likely due to the change in the atomic positions of the oxygen atoms when transitioning from the monoclinic to the rutile phase. During this transition there is a lattice parameter change of ~ −1.2 Å from a = 5.75 Å in the monoclinic phase to a = 4.55 Å in the rutile phase^15^.

Finally, we sought to compare our bulk VO_2_ simulations to a VO_2_ sample grown on TiO_2_(001). Upon examination of the VO_2_ film prior transition we find in (Fig. 2d) a plausible superposition of the monoclinic and rutile phases with a greater emphasis on the streak locations of the rutile phase. Especially in the case of (Fig. 2d), the non-transitioned locations have a distinctly rutile like pattern that suggests structural strain toward the rutile phase. Thus for (Figs 2d and S2c), we have interlaid the monoclinic and the rutile simulations to make the microstructure more apparent enabling fruitful comparison with experiment. This combined with a 0° rotation asserts that there is microstructural strain toward the rutile phase in the non-transitioned VO_2_ grown on TiO_2_. We then heated the sample to 100 °C to allow full IMT, which for this sample is ~42 °C where film substrate strain effects are known to play a key role in lowering the transition temperature^12,18^.

Once we heated the sample, we compared the simulations with the experimentally determined RHEED patterns. Here we find that the patterns for the 0° and the 90° azimuth positions, observed in (Fig. 2e), have several streaks well matched to the simulations. In comparison to the monoclinic phase, the center streak is missing but the four streaks that make up the “rectangular” shape of the pattern still persist with a slightly larger streak spacing than that of VO_2_ on c-Al_2_O_3_. We also see this in (Fig. S2d) the 45° azimuth position for the diffraction pattern, along with similar streaks seen in the simulation. Also, this pattern now exhibits a slight slant to the locations of the streak that would occupy the same lattice plane. This is likely due to the structural strain on the atomic locations on the lattice, namely the oxygen locations. Slight displacements off the main axis of the lattice could also cause shifts to the pattern, which is the case for the 45° direction. This slight displacement is also visible in the 0° and the 90° azimuth in-plane directions, but the shifts are lesser here since these directions represent less sensitive directions to oxygen displacements.

We examined the RHEED pattern through the transition to see changes due to the IMT. In order to do this, we heated the sample in 5 °C increments from 25 °C to 100 °C recording the diffraction pattern at each increment in agreement with the previous VO_2_ on c-Al_2_O_3_ sample. Upon ramping through the temperature increments, we found a marked change that occurred at the critical temperature. We observed the disappearance of the center streak in the rutile state that was apparent in the non-transitioned phase as demonstrated by (Fig. 2f) and Supplementary Video S2. This change occurs abruptly upon the IMT. As previously described, due to the strong dependence of the streak patterns to the oxygen location and the larger degree of structural strain on the sample, this large pattern adjustment agrees with the expected strained monoclinic structure as previously discussed.

The structural characterization across the transition for these samples provides a suitable framework for subsequent characterization of the photo responsivity of the same samples described below.

### Photocurrent analysis

**Figure 3:**
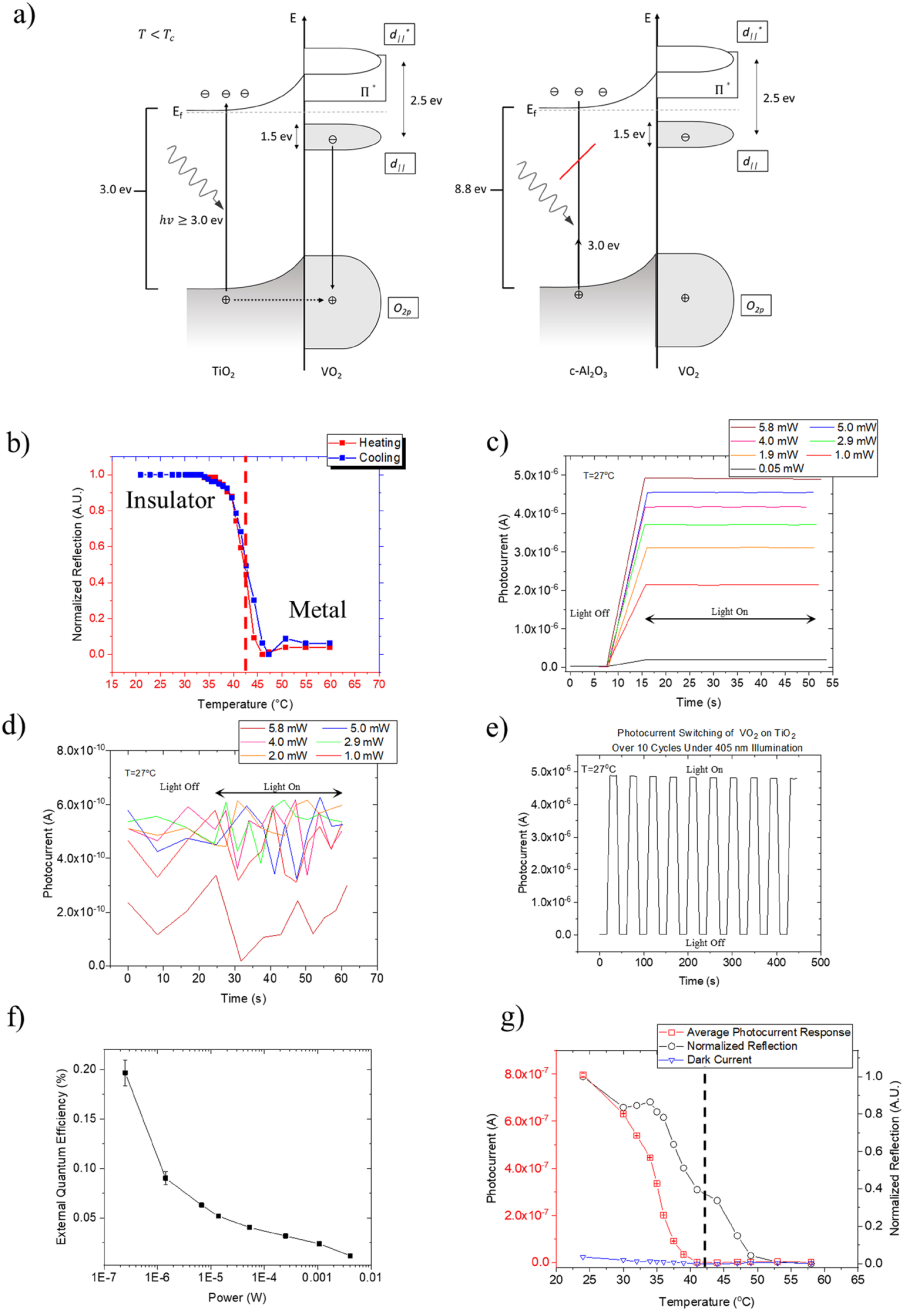
The electronic structure of VO_2_ on TiO_2_ and c-Al_2_O_3_ as well as studies of the photoelectric properties of VO_2_ on TiO_2_. (**a**) Schematic band diagram of the hole transfer mechanism for VO_2_ on TiO_2_ and c-Al_2_O_3_ where the photon energy is demonstrated for the VO_2_ on TiO_2_ as being sufficient to excite carriers in the TiO_2_ layer and insufficient in the case of VO_2_ on c-Al_2_O_3_. Where the hole transfer is designated by the segmented line and the carrier movement is designated by the solid lines^6,19^. (**b**) Reflectivity measurements with a 405 nm diode laser illuminating VO_2_ deposited on TiO_2_(001) as the sample underwent a thermally induced IMT via heating and subsequent cooling where the dashed line indicated transition temperature. (**c**) The photocurrent switching of VO_2_ on TiO_2_(001) upon solely 405 nm illumination as the laser power was varied. The light was held on for 40 seconds post switching. (**d**) The same photocurrent switching measurement of VO_2_ on c-Al_2_O_3_(0001) where the 405 nm laser power was varied. The light was held on for 40 seconds post switching. (**e**) The photocurrent switching cycle for VO_2_ on TiO_2_(001) upon 405 nm illumination switching over 10 cycles. (**f**) External quantum efficiency measurement for VO_2_ on TiO_2_ as varied with laser power. (**g**) The average photocurrent and optical response of VO_2_ on TiO_2_(001) as the sample was thermally ramped through the optical transition where the 405 nm laser power was 1 mW through the thermal ramping where the dashed line indicates transition temperature.

As demonstrated by (Fig. 3a) the VO_2_/TiO_2_ band structure illustrates that such film substrate heterostructure as a promising candidate for an efficient near-UV to deep UV photo-sensor; which is of interest due to lack of efficient, low power photodetectors in this spectral range. Thus, we examined how the photocurrent produced in these samples under 405 nm illumination is affected through the IMT. Due to the close match between the energy of the incoming photon and the TiO_2_ substrate band gap energy, the electron carriers are excited from the O_2p_ band into the conduction band of the TiO_2_ substrate. By exciting carriers in the TiO_2_ substrate, the holes left behind in the TiO_2_ O_2p_ band are then transferred to the VO_2_ O_2p_ band leading to carriers from the d_||_ band of VO_2_ moving to the O_2p_ band thus inducing a photocurrent^6^. Worth noting is that strain effects on the structure of the VO_2_ could result in adjustments to the band structure shown. We anticipate that the d_||_ and d_||_^*^ bands of the VO_2_ could exist closer together energetically resulting in a more metallic electronic structure as the lattice is strained toward the rutile phase.

This photocurrent is only possible in the monoclinic phase as once VO_2_ undergoes the phase transition to the rutile phase the band gap collapses and it takes on characteristics of a metal, therefore producing no photocurrent. To this end, we first demonstrated the optical response of VO_2_ on TiO_2_ as it undergoes the thermal IMT. This optical transition is demonstrated in (Fig. 3b) where a decrease in voltage is correlated to a decrease in resistivity due to the IMT. We then investigated if a photocurrent could be produced under solely illumination of 405 nm light, chosen because its energy (~3.06 eV) is close to the band gap of TiO_2_ is (~3.0 eV)^20^ thus providing optimal photon energy to properly excite carriers in the TiO_2_ substrate as demonstrated by (Fig. 3a). In (Fig. 3c) we show that the 405 nm laser produces a clear photocurrent, and its magnitude is correlated with the laser power. We also observed a reliable switching of the photocurrent switching through multiple on/off cycles, demonstrating its survivability as demonstrated by (Fig. 3e). We additionally examined the quantum efficiency of the TiO_2_ samples under various laser powers in (Fig. 3f). The external quantum efficiency was calculated via

1 $$EQE=\frac{hc{\rm{\Delta }}I}{e\lambda P}$$

Where Δ*I* is the photoexcited current, *P* is the total light power irradiated on the VO_2_, *h* is Planck’s constant, *e* is electron charge, and *λ* is the excitation wavelength. We find that as we decrease the laser power on the surface of the VO_2_ the quantum efficiency exhibits a 2000% increase through the laser power reduction as demonstrated by (Fig. 3f). This is potentially due to the carrier excitation in the substrate being inefficient at high powers due to the large influx of photons and the number of available carriers is less than the number of photons available to excite said carriers; as the laser power is decreased the photon to carrier excitation matching (and thus the overall carrier excitation per photon absorbed) is improved, in turn improving the overall quantum efficiency. We also attempted to measure the photocurrent of VO_2_ on c-Al_2_O_3_(0001) upon 405 nm illumination. Since the 405 nm light’s photon energy is insufficient to excite carriers in c-Al_2_O_3_(0001) across its ~8.8 eV^21^ band gap,_,_ we see limited to no photocurrent in the VO_2_ film as shown in (Fig. 3d). We in fact found that the produced photocurrent was 4 orders of magnitude smaller and on the order of the dark current, the small current that exists in photosensitive devices when in absence of photons, leading to the lack of defined features in the corresponding graph as opposed to VO_2_ on TiO_2_(001).

Finally, we examined the photocurrent produced upon heating the sample and correlated the transition with the photocurrent. As demonstrated in (Fig. 3g), we observe that as the sample is heated, a marked change in the photocurrent produced is noted, approaching zero until it eventually reaches zero. This reduction in photocurrent is due to the IMT where the collapse of the band gap results in VO_2_ becoming metallic preventing the production of a photocurrent as previously described. Here our reflected optical signal tracks the phase change optically through the transition, where a reduction in optical response is correlated with the phase change from monoclinic to rutile. However, we note that the photocurrent reduces to zero before the optical transition is complete. This finding would suggest that the migration of carriers in VO_2_ is greatly reduced before the band gap is fully collapsed. One possible explanation follows from earlier descriptions of the nucleation of the metallic phase upon heating where localized puddles of the metallic phase in the VO2 sample become so prevalent such that hole migration from the substrate to the film is substantially impeded preventing the production of photocurrent before the film is fully transitioned to the metallic phase^1,4,22^.

Thus, local “puddle” formation results in loss of effective carrier movement before the sample has fully transitioned such that a global surface current is no longer possible reducing the photocurrent to zero although the surface of the sample is not fully phase transitioned. This “puddling” across the thermally induced IMT in VO_2_ has been extensively studied and reported^1,4,22^.

## Discussion

We have shown that epitaxially grown VO_2_ on both c-Al_2_O_3_ and TiO_2_(001) by pulsed DC sputtering exhibit good crystal character and a low degree of mosaicity. They also exhibit a characteristic surface morphology with terracing plateaus due to strain. We have been able to accurately compare simulations of VO_2_ RHEED patterns consistent with our experimental data on the films that we have studied. We have also presented a first of its kind RHEED temperature transition analysis for VO_2_ in which we were able to analyze and conceptualize the structural phase transition of VO_2_ on both c-Al_2_O_3_ and TiO_2_(001), evidencing the dynamical changes that VO_2_ undergoes through its critical transition temperature.

We also determined the photocurrent switching of VO_2_ on TiO_2_(001) and c-Al_2_O_3_(0001) at 405 nm and saw a stark reduction in photocurrent for VO_2_ grown on c-Al_2_O_3_(0001). This reduction in photocurrent is likely a product of the high resistivity and large substrate band gap as well as VO_2_ films with much rougher surface, where roughness also contributes to scattering effects resulting in lower carrier efficiency thus reducing the photocurrent. We have also demonstrated a marked increase in the quantum efficiency of the VO_2_ on TiO_2_ with decreasing laser power seeing as large as a 2000% difference in quantum efficiency from 4.6 mW to 250 nW laser power. Finally, we have demonstrated how the IMT influences the produced photocurrent under illumination determining that as the VO_2_ transitions through the IMT to the metallic state there is a significant reduction of produced photocurrent, reaching zero value once the global state of the surface is metallic hindering production of photocurrent for VO_2_.

## Methods

### Sample growth

Epitaxial VO_2_ films were sputtered-deposited on one side polished (1-sp) c-Al_2_O_3_(0001) and 1-sp TiO_2_(001) substrates in an ultrahigh vacuum (UHV) deposition system with a base pressure ~3.0 × 10^−7^ Torr range. The films were deposited via reactive D.C. pulsed magnetron sputtering with a vanadium target of 99.95% purity in a 90% Ar and 10% O_2_ environment at 550 °C. Prior to deposition the substrates were annealed *in-situ* for 30 minutes at 600 °C to de-gas and recrystallize the top-most surface layers on each substrate. The target voltage was periodically pulsed to a positive charge to eject the excess insulating oxide layers that usually poison the surface of the target during sputter deposition. Growth rates were determined via X-Ray Reflectivity (XRR) in calibration samples. The films were evaluated via Reflection High-Energy Electron Diffraction (RHEED), Atomic Force Microscopy (AFM), X-Ray Diffraction (XRD), and Van der Pauw 4-point probe (VDP) measurement.

### Rheed, AFM, XRD, and photocurrent characterization

RHEED was used to determine the crystallographic structure of the film surface. This technique utilizes glancing incident angle geometry for an electron beam on the probed surface allowing the beam to sample mainly the top-most layers of the film^13,14^. Thus, RHEED enables qualitative and quantitative descriptions of the in-plane surface microstructure. The space groups for the two phases of VO_2_ across the IMT are P4_2_/mmm(136) for rutile with lattice spacing a = 4.55 Å, c = 2.88 Å, and P2_1_/c (14) for monoclinic with lattice spacing a = 5.75 Å, b = 5.42 Å, c = 5.38 Å (we note that the drastic change in c lattice spacing is due to a doubling of the unit cell along the c direction)^15^. This information allows comparisons of various experimental diffraction patterns to predicted models for both phases. This technique also allows us to map the crystallographic changes through the thermally induced IMT.

The surface morphology of the samples was characterized via *ex-situ* AFM. The AFM images were collected using a Nanotec Cervantes AFM instrument. The AFM was operated in non-contact mode with an AppNano ACTA AFM tip with nominal tip diameter ~10 nm. The software WSxM from Nanotec was used for analysis of the AFM images^23^. All images were flattened in the WSxM program and the images were processed via the root mean squared (RMS) analysis tool in the program. The RMS roughness gives an average estimate of the surface roughness of the film, and this quantitative description can be paired to the qualitative description obtained from RHEED.

The microstructure of the films throughout their full thickness and out of plane crystallographic information was determined via XRD. The instrument used was a four-circle diffractometer with a quasi-monochromatic CuK_alpha_ (λ = 1.5406 Å) beam. This technique was used to determine the out of plane lattice parameter, grain size^24^, and mosaicity of the films.

The electrical and quantum efficiency information was determined with an MMR Technology Variable Temperature Microprobe System (VTMP) and a 405 nm diode laser for the VO_2_ grown on TiO_2_ as well as a 1520 nm laser for the VO_2_ grown on c-Al_2_O_3_. The sheet resistance was determined via the Van der Pauw method^25^. The samples were illuminated with a 405 nm laser and the produced photocurrent was measured. The samples were then thermally ramped through the IMT and both the photocurrent, using the VTMP, and the optical response, using a 405 nm photodetector, were recorded simultaneously for each sample.

### RHEED simulations

The simulations for this experiment were carried out using the RHEEDsim MATLAB program^26^. This software utilizes the atomic positions of the atoms in VO_2_ films as well as TiO_2_ and c-Al_2_O_3_ substrates determined from the space groups of the respective molecules. The program utilizes a kinematic approach for single scattering events, and then approximates the Ewald sphere to a planar surface to compute streak intensities. Additionally, intensity modulation extends to within the z direction^26^. The form factors for atomic scattering were determined via NIST standards for X-ray form factor, attenuation, and scattering tables^27^. This simulations yield realistic representations for comparison to experimentally determined RHEED diffraction patterns.

## Supplementary information

Supplementary Information for Structural and Photoelectric Properties of Epitaxially Grown Vanadium Dioxide Thin Films on c-Plane Sapphire and Titanium Dioxide

VO<sub>2</sub> on c-Al<sub>2</sub>O<sub>3</sub> Insulator to Metal Transition Imaged with RHEED

VO<sub>2</sub> on TiO<sub>2</sub> Insulator to Metal Transition Imaged with RHEED
