# Supplementary material for: Structural and Photoelectric Properties of Epitaxially Grown Vanadium Dioxide Thin Films on c-Plane Sapphire and Titanium Dioxide
Source: Sci Rep. 2019 Jun 27;9:9362. doi: 10.1038/s41598-019-45806-8 (PMC6597701; doi:10.1038/s41598-019-45806-8)
Supplement: Supplementary file 1 — Supplementary Information for Structural and Photoelectric Properties of Epitaxially Grown Vanadium Dioxide Thin Films on c-Plane Sapphire and Titanium Dioxide [file 41598_2019_45806_MOESM1_ESM.doc]

**Supplementary Information for Structural and Photoelectric Properties of Epitaxially Grown Vanadium Dioxide Thin Films on c-Plane Sapphire and Titanium Dioxide**

*Jason A. Creeden^1*^, Scott E. Madaras^1^, Douglas B. Beringer^1^, Melissa R. Beebe^1^, Irina Novikova^1^, and R. Ale Lukaszew^1^*

^1^ William & Mary Department of Physics, Williamsburg, Virginia 23187, USA

[^*^jacreeden@email.wm.edu](mailto:*jacreeden@email.wm.edu)


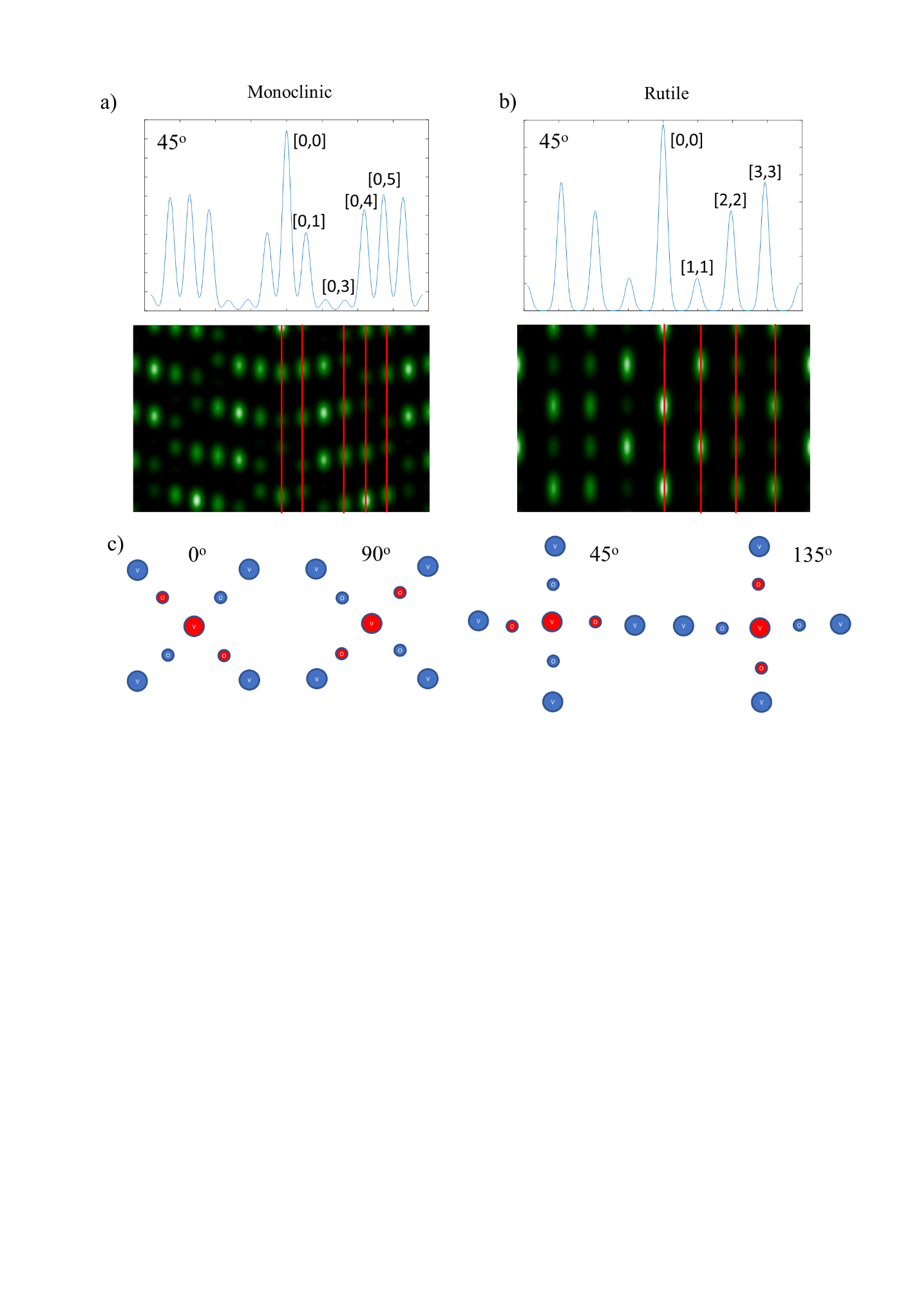


**Figure S1: The experimental XRD, AFM, and simulated RHEED patterns of VO_2_ on c-Al_2_O_3_ and TiO_2_.** a) The simulated RHEED patterns of the 45^o^ rotation for the monoclinic phase of VO_2_ where the streak intensity patterns are recorded on top and the simulated diagrams are reported on bottom with the in plane lattice planes are recorded for each. b) The simulated RHEED patterns of the 45^o^ rotation for the rutile phase of VO­_2_ where the placement of the plots is the same as the previous monoclinic phase. c) The orientations of the vanadium and oxygen atoms through the rotations of one unit cell where the first two surface layers of atoms are shown in the c-direction where the red layer is displace ~1.44 nm below the blue layer.

We demonstrate the 45^o^ azimuthal rotation RHEED simulation for VO_2_ in both the monoclinic and rutile phases shown in Figure S1a-b). Additionally, in Figure S1c) we show the lattice positions of the vanadium and oxygen atoms through various azimuthal rotations. We also demonstrate the azimuthal rotations in the experimental RHEED patterns for VO_2_ through the transition as shown in Figure S2a-d) that were not shown in the primary manuscript.


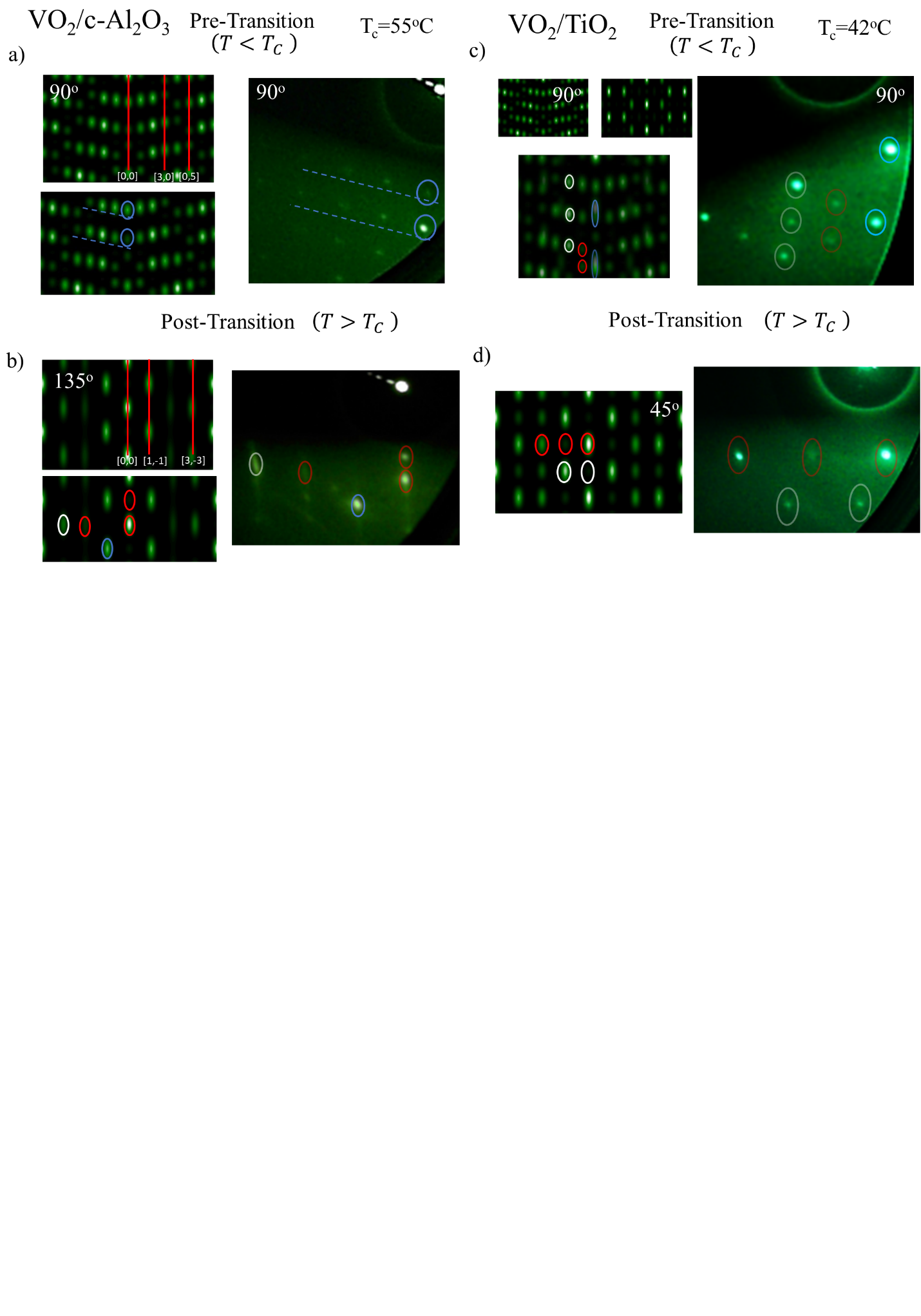


**Figure S2: The simulated and experimentally determined RHEED patterns for VO_2_ on c-Al_2_O_3_(0001) and VO_2_ on TiO_2_(001). For VO_2_ on c-Al_2_O_3_(0001):** Simulated and experimental RHEED patterns of VO_2_ azimuthal rotations and for the two phases. a) The left two images are simulations of the 90^o^ rotation for the monoclinic phase of VO_2_; the top image denotes the 2-D lattice planes for the streak patterns while the bottom image has streaks highlighted. The right image is the experimentally determined RHEED pattern the highlighted streaks correspond to the simulated image. b) The plot orientation is maintained for where the simulated images are shown at left and experimental images at right for the 135^o^ rotation for the rutile phase of VO_2_. **For VO_2_ on TiO_2_(001):** c) The 90^o^ rotation for the VO_2_ on TiO_2_ pre-transition where the top leftmost two images are simulations of the 90^o^ rotation for the monoclinic phase (left) and rutile phase (right) of VO_2_*.* The bottom left image is a superimposed image of both simulated images. The right image is the experimentally determined RHEED pattern for VO_2_ on TiO_2_(001) where the highlighted streaks correspond to the simulated images. d) The left image is the simulated 45^o^ rotation for rutile phase VO_2_ and the right image is the experimental pattern for the rutile phase VO_2_ grown on TiO_2_(001). (Note: All images have been rotated 45 degrees and the contrast has been increased for ease of streak identification)
